# Harnessing the innate immune system and local immunological microenvironment to treat colorectal cancer

**DOI:** 10.1038/s41416-019-0441-6

**Published:** 2019-04-02

**Authors:** Jakob Nikolas Kather, Niels Halama

**Affiliations:** 1 Department of Medical Oncology and Internal Medicine VI, National Center for Tumor Diseases, University Hospital Heidelberg, Heidelberg, Germany; 2 German Translational Cancer Consortium (DKTK), Heidelberg, Germany; 3 Applied Tumor Immunity, German Cancer Research Center (DKFZ), Heidelberg, Germany; 4 Institute for Immunology, University Hospital Heidelberg, Heidelberg, Germany; 5 Department of Translational Immunotherapy, German Cancer Research Center (DKFZ), Heidelberg, Germany; 6 Helmholtz Institute for Translational Oncology (HI-TRON), Mainz, Germany

**Keywords:** Immunotherapy, Tumour immunology, Cancer immunotherapy

## Abstract

Significant progress in the development of new immunotherapies has led to successful clinical trials for malignant melanoma and non-small cell lung cancer; however, for the majority of solid tumours of the gastrointestinal tract, little or no progress has been seen. The efficacy of immunotherapies is limited by the complexities of a diverse set of immune cells, and interactions between the tumour cells and all other cells in the local microenvironment of solid tumours. A large fraction of immune cells present in and around solid tumours derive from the innate arm of the immune system and using these cells against tumours offers an alternative immunotherapeutic option, especially as current strategies largely harness the adaptive arm of the immune system. This option is currently being investigated and attempts at using the innate immune system for gastrointestinal cancers are showing initial results. Several important factors, including cytokines, chemotherapeutics and the microbiome, influence the plasticity and functionality of innate (myeloid) cells in the microenvironment, and this complexity of regulation has limited translation into successful trials so far. In this review, current concepts of the immunobiology of the innate arm in the tumour microenvironment are presented in the context of clinical translation.

## Introduction

The local microenvironment of solid tumours is a complex system comprising cells of the immune system, fibroblasts, endothelial cells and many other cell types.^1–3^ Immune cells have different roles in the microenvironment, including pro-tumorigenic^4^ and anti-tumorigenic^5^ functions. Conceptually, the immune system can be divided into two major parts: the innate arm, which consists of an older evolutionary defence strategy, and the adaptive/acquired arm, which creates adaptive immunological memory. Although both arms of the immune system can be distinguished conceptually, they are functionally interlocking and thus heavily influence each other.^6^

One of the hallmarks of cancer is chronic inflammation,^7^ which fuels and sustains disease progression and neoplastic transformation;^8^ for colorectal cancer (CRC), this is most obviously evidenced for inflammatory bowel disease (IBD), which carries a significant risk of malignant transformation.^9^ Different sources of this inflammatory process have been identified, including persistent infections and sterile inflammation; for both of these sources, cells of the innate immune system can be the primary effector type. Although the extent of the individual contribution of these various innate cells to the primary inflammatory response is not precisely known, it is clear that dynamic changes in the microenvironment follow a specific pathway that is exploited by the tumour. The tumour-promoting pathway begins with continuous inflammatory signals provided by the tumour itself or via the host’s own immune system to eradicate the tumour cells. Inflammatory signals can consist of apoptotic cells, damage-associated molecular patterns, free DNA molecules, heat shock proteins and Toll-like receptors (TLRs)/ligands or cytokines, which may lead to the futile activation of immune cells.^8,10–12^ Subsequent chemokine production leads to an influx of more immune cells that can drive further activation or inactivation of immunological processes and can end up fuelling tumour growth and dissemination.

The main components of the innate immune system are physical epithelial barriers, phagocytic leukocytes (such as granulocytes and macrophages), dendritic cells, natural killer (NK)/innate lymphoid cells and circulating plasma proteins. This arm of the immune system is present in all tissues; however, its role in immunotherapy is poorly understood.^7,13^ Our understanding of the innate arm of the immune system and its complexities has been limited by the inherent fundamental functional differences between the immune system in animal models and in humans.^14–17^ Another factor contributing to our limited understanding concerns difficulties in identifying innate cell subsets in the local microenvironment through unambiguous surface markers reflecting functional states of cells; e.g., NK cells were long thought to be an influential factor in CRC and breast cancer, but analyses showed an unexpected absence of these cells from these tumours,^18–21^ despite the presence of chemokines and adhesion molecules. The different origins of myeloid cells^22^ and specific differentiation programmes for myeloid subtypes^23^ add another layer of complexity in regulation.

**Fig. 1:**
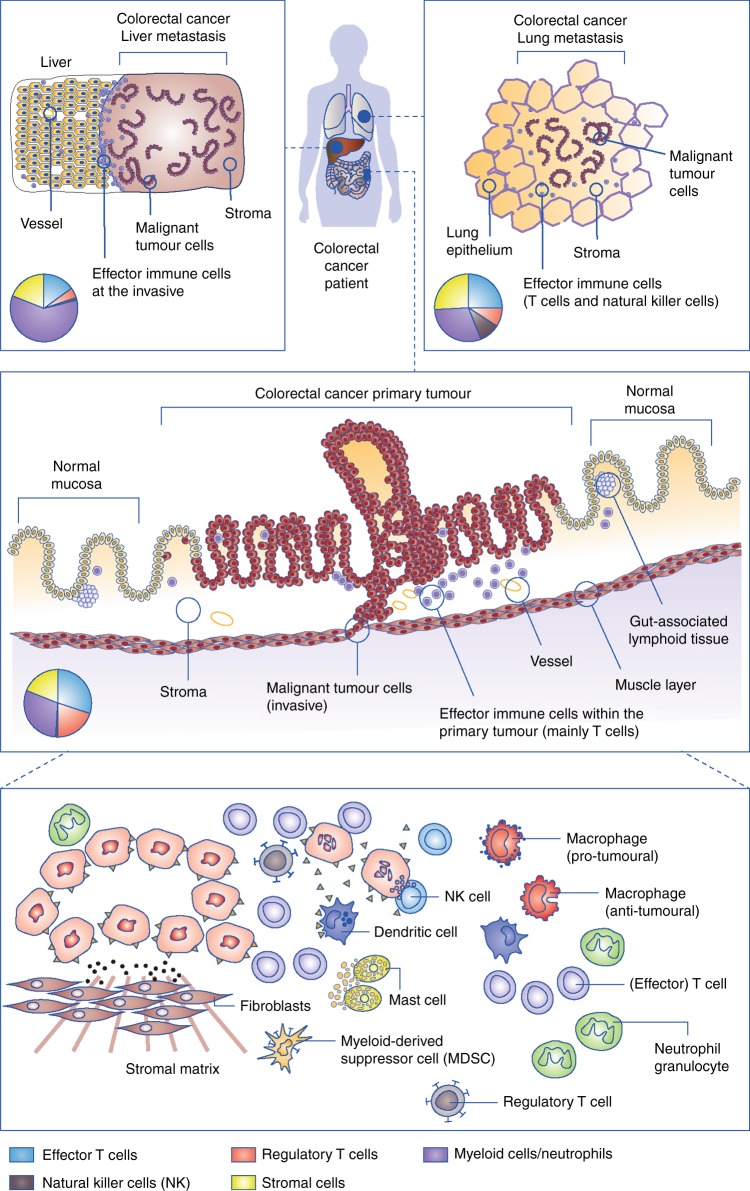
Overview of the composition of the immunological microenvironments in different lesions (primary vs. lung and liver metastases) of colorectal cancer (CRC). The pie charts provide examples of immune cell composition within the local microenvironment (data from Halama et al.^27^) to highlight organ-specific heterogeneity. The lower panel provides an overview of the key immune cells that are present in the immunological microenvironment

Looking into the composition of immune cells in solid tumours, myeloid cells can form a significant proportion of cells in the microenvironment, outnumbering lymphocytes and occasionally even the tumour cells themselves.^24^ Furthermore, fibroblasts and other mesenchymal cell types form an important component of the microenvironment,^25^ influencing hypoxia, migration of immune cells and the metastatic behaviour of tumour cells.^26^ This heterogeneity of the immune cell phenotypes present in the microenvironment across different cancer entities and metastatic sites (Fig. 1)^27^ is just one hurdle to overcome for successful immunotherapy; specific cellular distribution patterns (e.g., the exclusion or the dense infiltration of T cells in immune-excluded tumours), functional plasticity and organ-specific functions form a complex set-up that is further complicated by the influence of the tumour cells, all of which pose a challenge to therapeutic approaches. The complex interplay between the innate and adaptive arms of the immune system is, of course, also of relevance for therapeutic effects.

In contrast to the belief that the local immunological microenvironment of solid tumours is a chaotic and dysregulated site, we propose that it is a site with a specific pro-tumoural regulation. This review will discuss the immunobiology of the innate arm of the immune system in the microenvironment of CRC and the therapeutic potential of innate immune cells (with the exception of dendritic cells, see refs. ^28–30^) for immunotherapy.

## The local microenvironment in CRC

Many publications have reported on the frequencies of immune cell subpopulations in different solid tumours and an association between immune cell density and clinical course has been shown by different groups for CRC.^7,31–36^ For many solid tumours, a high density of infiltrating T-effector cells is associated with a good prognosis and conversely a high density of myeloid cells is associated with a poor prognosis. Interestingly, the subpopulations relevant to tumour response and progression can vary between different cancers.^37^ Fridman^38^ proposed the concept of an ‘immune contexture’, which suggests that different compositions of immune cells and signalling molecules have specific roles in each cancer entity.

In CRC, the adaptive arm specifically has been shown not only to learn to recognise tumour cells but also to contribute greatly to the course of the disease. The presence of effector T cells in the local microenvironment is typically regarded as a sign of inflammation, whereas the presence of regulatory T cells is regarded as a sign of immunosuppression. High effector T-cell density is associated with a clear prognostic advantage across several different cancers; in CRC, the presence of effector T cells is linked to a good prognosis for both the primary tumour and in metastatic settings.^39–44^ The role of FOXP3 + regulatory T cells, however, is debated.^45^ Normally, regulatory T cells are regarded as immunosuppressive, abrogating an effective immune response against the tumour; however, in CRC, higher densities of these regulatory T cells are associated with a better prognosis, opposing the negative association of FOXP3^–^ T lymphocytes in other cancer types. In metastatic liver lesions of CRC, the composition of the local microenvironment is mainly driven by chemokine gradients and cytokines, with only low numbers of NK cells or regulatory FOXP3 + T cells present.^46^ A small subgroup of patients with CRC have microsatellite-instable (MSI) tumours and show a massively increased presence of infiltrating adaptive immune cells (i.e., lymphocytes), with numbers more than twice as high as the average density in microsatellite-stable (MSS) CRC.^47,48^ In patients with MSI tumours, faulty DNA repair proteins give rise to more immunologically relevant mutations and produce a better control of the tumour through the immune system, which correlates with a better prognosis in these patients. Whereas MSI tumours respond well to immunotherapy, MSS CRC does not respond positively.

The role for B cells in the microenvironment is highly controversial in CRC, with data from quantification and localisation studies showing no clear significant association with clinical course in multivariable analysis.^49^ Future analyses should address the interplay between B cells and other innate immune cells in CRC.^50^ The presence of B cells and T cells together, as occurs in tertiary lymphoid structures, has been confirmed and analysed in CRC. In short, the presence of these tertiary lymphoid structures indicates a more favourable prognosis, owing to the increased infiltration of immune cells. There is also data, however, that associates tertiary lymphoid formation with BRAF mutation.^51–54^

## Current immunotherapeutic approaches for CRC

Despite the multiple avenues that have been investigated to achieve tumour control, immunotherapy for CRC has so far largely failed to show clinically meaningful effects. Classic vaccination strategies have not shown significant effects; it remains to be seen whether more personalised approaches (e.g., mutanome vaccines based on sequencing efforts) will lead to effective vaccinations.^55–58^ Chimeric antigen receptor T-cell approaches have shown some positive effects; however, these were limited by severe toxicity or by efficacy limited to a specific mutation.^59,60^ A small subgroup of patients with mismatch-repair deficient (MMRd)/MSI CRC have shown good responses to checkpoint inhibition (e.g., via anti-PD-1, anti-PD-L1 or anti-CTLA-4: all three targets are present in the microenvironment)^61^ and this has led to the approval of anti-PD-1 antibodies for MSI CRC. This responsiveness to checkpoint inhibition most likely stems from the strong presence of T cells within the microenvironment in this subtype of CRC, as similar successes have not yet been reported for MSS CRC using checkpoint inhibition on its own.^62–66^ Systematic analyses of the mutational burden in MSS CRC has identified a subgroup of patients with high mutational burden but without MSI^67,68^; whether these patients would benefit from a systemic therapy with checkpoint inhibition remains unclear. Interestingly, a combined approach using chemotherapy (FOLFOX plus bevacizumab, NCT01633970) and anti-PD-L1 has shown some clinical effects in patients with (MSS) CRC.^69^

Determining why checkpoint inhibition does not work in patients with MSS CRC is a key question for immunotherapy. Resistance mechanisms in solid tumours are currently being systematically analysed; these mechanisms include induction of T-cell anergy via metabolic deprivation, inhibition of effector T-cell migration into the tumour tissue, T-cell inactivation via specific receptor–ligand interactions and barrier functions of the stroma, among others.^70,71^ Recent data suggest that some resistance mechanisms might be mediated by macrophages.^72^ In a broader approach, chemotherapy was combined with immunomodulation in the GOLFIG trials, in which a combination of gemcitabine, oxaliplatin, folinic acid, fluorouracil, interleukin (IL)-2 and granulocyte-macrophage colony-stimulating factor (GM-CSF) was administered.^73,74^ The initial data looked suggestive of enhanced efficacy; however, this approach was not continued due to recruitment problems and a modified protocol is being investigated (FOLFOXIGIL trial, NCT03222089). Broader still, histone deacetylase inhibitors have shown efficacy against lung cancer and other cancer entities, by inducing the reversal of T-cell exhaustion, among other means^75^; however, the effect of histone deacetylase inhibitors on macrophages and other immune cells in CRC is unclear.^76^

### Other modulators of the immune microenvironment

Although the role of chronic inflammation as a driver for tumorigenesis is widely accepted (as mentioned above, chronic inflammation in IBD is associated with a higher risk of CRC), the role of inflammation and the immune system in non-IBD-mediated tumorigenesis is still unclear, especially as the role of non-steroidal anti-inflammatory drugs such as aspirin is still debated.^77^ Clinically, lower incidence rates of CRC and increased survival are associated with continual aspirin intake,^78–80^ but the molecular basis for this observation is not entirely clear^81^; however, the mutational status of the *PI3KCA* gene in tumour cells has been identified as one possible factor for the impact of aspirin.^82–84^ From the immunological standpoint, it is also not so clear. Although associations between the composition of the immunological microenvironment and aspirin intake have been observed,^85^ aspirin’s precise immunological mode of action remains unknown. More globally, we need to better understand the mechanisms of early carcinogenesis and the influence of adaptive and innate immunity at this stage, as well as the effect of other modulators of the immune system. For example, the level of vitamin D reportedly shows an association with the occurrence of CRC^86^ and clearly influences the composition of the local immunological tumour microenvironment^87^—higher plasma levels of vitamin D are associated with fewer tumours with higher T-cell infiltration. Precisely, how vitamin D influences monocytic cells in vivo remains unclear but differential modulation of the molecular response of monocytes, macrophages and dendritic cells to innate immune stimulation has been observed.^88^

Along the same lines, fatty acids have a profound role in modulating the local tumour microenvironment and the innate arm of the immune system.^89^ The association of a high intake of fibre with the suppression of inflammation is just one example of how nutrition can alter the local microenvironment in CRC^90,91^ and brings together the complexity of the areas of immunology and the microbiome.^92,93^ The influence of the microbiome on the innate immune system in CRC will be discussed below.

## Immunotherapy for CRC: targeting innate immune cells

The composition and density of myeloid and non-myeloid immune cells in the CRC tumour microenvironment is surprisingly stable over time.^94^ Nevertheless, the plasticity of human myeloid cells and their lack of high-precision markers make it difficult to quantify, annotate and functionally characterise these cells. Their localisation and density together form an intrinsic network that reflects the activation and functionality of these cell populations, and requires sophisticated detection and quantification algorithms.^95,96^ Below we describe the key cells involved in innate immunity and current strategies to target them as a therapeutic approach to CRC.

### Macrophages

**Fig. 2:**
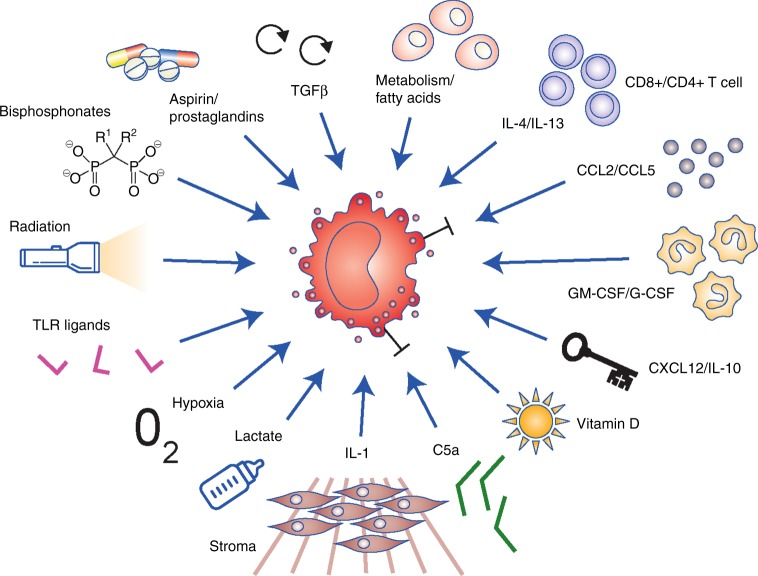
Macrophage cell plasticity also translates to functional plasticity. Functionally relevant signals from within the microenvironment can influence whether macrophages adopt an anti-tumour type I (M1) phenotype or an immunosuppressive type II (M2) phenotype, or any intermediary complex phenotype. Different combinations of these signals can further dynamically affect macrophage differentiation and functionality

Macrophages are among the most abundant cells within the CRC microenvironment and, together with myeloid-derived suppressor cells (MDSCs), they perform a diverse set of roles that includes skewing and suppressing adaptive immunity, orchestration of tissue repair and damage regulation, promotion of immunosuppression, modulation of the response to immunogenic cell death (‘adjuvanticity’), effector functionality against tumour cells and the mediation of abscopal effects.^97^ Macrophage plasticity is an important feature and the ability of different interventions (e.g., chemotherapy, radiation, etc.) to induce a rapid change in their function can be characterised, e.g., by changing from an immunosuppressive type II (M2) macrophage to an anti-tumour type I (M1) phenotype (Fig. 2). The net anti-tumoural effect can vary greatly (Table 1), although nearly all forms of intervention lead to modulation of macrophages in the tumour microenvironment. The factors that mediate this plasticity are highly diverse: cytokine and chemokine signals (IL-1, IL-4, IL-13, C–C motif chemokine ligand 5 (CCL5), CCL2, GM-CSF, CXCL12, IL-10, etc.) through to inflammation signals (e.g., prostaglandins, TLRs and ligands, complement system components), drugs (e.g., bisphosphonates) to metabolic and endocrine signals (e.g., fatty acids, lactate or vitamin D) and all forms of tissue stress (e.g., hypoxia, radiation) can modulate and alter macrophage functionality and consequently influence the neighbouring tumour.^24,97–101^ This influence can be both positive and negative, in an effect that is typically referred to as the ‘Yin–Yang’ of myeloid cells, whereby anti-tumoural effects as well as resistance to an intervention (e.g., chemotherapy with fluorouracil or bevacizumab) is mediated by the same cell type.^24,102–105^ Not only for CRC but also for all other cancer types, the functionality defining signals and signal combinations for macrophage modulation are starting to emerge, and with them the opportunity to design interventions.^107^ However, the precise role(s) of the presence of macrophages with different phenotypes in CRC is still being investigated and so far no clear-cut picture emerges,^106^ especially with respect to the mutational status of the tumour.^108–110^

**Table 1:** Selected ongoing clinical trials targeting innate cells of the immune system in colorectal carcinoma

Pathway	Target	Efficacy in model systems/combinations	Clinical compounds	Clinical trials
Cell of primary interest: macrophage				
Recruitment	CD11b	Radiation, chemotherapy	Rovelizumab	
	CSF-1R	Single agent (GBM, PDAC, CRC), chemotherapy, radiation, angiogenesis inhibitors, checkpoint inhibition	PLX3397, AMG820, IMC-CS4/LY3022855, RG7155/RO5509554, PD-0360324, PLX108-01	NCT01596751; NCT01444404 NCT01349036; NCT01004861 NCT01346358; NCT02265536 NCT01494688; NCT02323191 NCT02777710; NCT01804530 NCT02554812; NCT02452424
	CCL2	Single agent (metastasis, PDAC)	Carlumab (CNTO888)	NCT00992186; NCT01204996
	Neuropilin-1	Angiogenesis inhibitors	MNRP1685A	NCT00747734; NCT00954642
	ANG2	Single agent (mammary), chemotherapy, angiogenesis inhibitors	Nesvacumab	NCT01271972; NCT01688960
	MIF	Single agent, chemotherapy	BAX69	NCT02448810
Polarisation	IL-4	Single agent (metastasis), chemotherapy, radiation	Pascolizumab	
	IL-4Ra		Dupilumab	
	IL-1	Single agent, chemotherapy (plus anti-VEGF)	Xilonix, anakinra	NCT01767857; NCT02090101
	IL-13	Chemotherapy	Lebrikizumab, tralokinumab, GSK679586,	
	FcγR	Chemotherapy	Rituximab (CD20), Ibrutinib (BTK), R788 (Syk)	
Repolarisation and activation	CCR5	Single agent (GI), chemotherapy, immunotherapy	Maraviroc	NCT01736813; NCT03274804
	CCR2+CCR5	Single agent, chemotherapy, immunotherapy	BMS-813160	NCT03184870
Function	IL-6		clazakizumab, olokizumab, siltuximab, sirukumab	NCT00433446; NCT00385827 NCT00841191
	IL-6R		tocilizumab, sarilumab	
	TNFα	Mitogen-activated protein kinase inhibitors	adalimumab, certolizumab, etanercept, golimumab, infliximab	
	CD47	Solid tumours	CC-90002, TTI-621	NCT02367196; NCT02663518
Activation	CD40	Single agent (PDAC), chemotherapy	CP-870,893	NCT00711191; NCT01456585 NCT02157831; NCT01008527 NCT02225002; NCT00607048 NCT01103635
	TLR agonists/antagonists	Single agent (maintenance), chemotherapy	MGN1703, VTX-2337	NCT02077868; NCT02650635
	MEK inhibition	Immunotherapy	Cobimetinib	NCT01988896
	Vitamin D/vitamin D binding protein	Single agent, chemotherapy, immunotherapy		NCT02052492; NCT02603757
Cell of primary interest: natural killer cell				
Cellular therapy	Cytokine-activated killer cells	Radiofrequency ablation		NCT02419677
	Dendritic and cytokine-induced killer cells	Single agent		NCT01839539; NCT02882659 NCT03047525; NCT03008499
	Cytokine-induced killer cells	Following surgery and chemotherapy		NCT02280278
	Cytokine-induced killer cells	Chemotherapy (S1 plus Bevacizumab)		NCT02487992
	Dendritic and cytokine-induced killer cells	Following surgery and radiation		NCT02202928
	Dendritic and cytokine-induced killer cells	Immunotherapy (anti-PD-1)		NCT02886897
Chimeric antigen receptor (CAR)	CAR-pNK cell	Single agent		NCT02839954
Co-stimulation/regulation	4-1BB	Cetuximab	Urelumab	NCT02110082
	CD27	Single agent	Varlilumab	NCT01460134
	KIR	Checkpoint inhibition (anti-PD-1, anti-CTLA-4)	Lirilumab	NCT01714739; NCT01750580
Cell of primary interest: fibroblast				
Cytokine modulation	FGF receptor	Single agent	Dovitinib	NCT01676714
	SDF1α/CXCL12	Single agent, immunotherapy	Olaptesed pegol	NCT03168139
Fibroblast targeting	(FAPα)	Single agent	F19	NCT00004042
Cell of primary interest: neutrophil granulocytes				
Inhibition of function	Arginase	Single agent, immunotherapy	CB-1158	NCT02903914
Reduction of neutropenia	Neutrophil granulocytes/bone marrow	Single agent, chemotherapy	MB-6	NCT02135887

Selected clinical trials and therefore a non-exhaustive list. *ANG2* angiopoietin-2, *CCL* CC motif chemokine ligand, *CCR* CC motif chemokine receptor, *CRC* colorectal cancer, *CSF-1R* colony-stimulating factor 1 receptor, *CTLA-4* cytotoxic T-lymphocyte-associated protein 4, *FAPα* fibroblast-activating protein α, *GBM* glioblastoma, *IL* interleukin, *IL-R* interleukin receptor, *KIR* killer immunoglobulin-like receptor, *MEK* MAPK/ERK kinase, *MIF* macrophage migration inhibitory factor, *NK* natural killer, *PD-1* programmed death ligand 1, *PDAC* pancreatic ductal adenocarcinoma, *SDF1α* stromal-derived factor 1α, *TLR* Toll-like receptor, *TNF* tumour necrosis factor, *VEGF* vascular endothelial growth factor

Different routes to target macrophages are being investigated in clinical trials (Table 1), ranging from macrophage depletion to macrophage repolarisation. As the name implies, depletion involves the destruction of macrophages in the tumour microenvironment, whereas the process of repolarisation tries to modulate the functional activity of the macrophages towards an anti-tumoural phenotype (i.e., cells that produce reactive oxygen species and interferons, or that phagocytose tumour cells). Strategies for abrogating macrophage recruitment to the specific organ or tumour tissue include the inhibition of chemokines and cytokines such as GM-CSF, vascular endothelial growth factor and CSF1, and modulation of pleiotropic cytokines such as macrophage migration inhibitory factor. Complement factor 5a also seems to have a role in the recruitment of myeloid populations into the tissue and into the tumour microenvironment. CCL2 is an example of a translational intervention aimed at modulating macrophage recruitment and data from a pancreatic ductal adenocarcinoma clinical trial combining CCL2 inhibition with chemotherapy are promising.^110^ Furthermore, CSF1–CSF-1R signalling is an important axis for the recruitment and generation of macrophage populations and extensive data from multiple groups have identified this signalling cascade as a central regulator of myeloid cell plasticity.^24,111^ Tumour responses were observed during clinical trials of a fully human CSF-1R antibody in patients with a rare diffuse-type giant-cell tumour;^112^ however, data from clinical trials in patients with malignant solid tumours show clear side effects and limited efficacy.^111^

Macrophage repolarisation therapy targeting the CCL5–CCR5 axis has been described in preclinical and clinical analyses for metastatic CRC.^46,114–116^ The chemokine effects of CCL5 on the migration of myeloid cells seems to have a minor role in this efficacy; rather, macrophage polarisation, with immediate effects on the production of interferon and reactive oxygen species, mediates these anti-tumoural effects and combination trials with checkpoint inhibitors are currently underway (NCT03631407 and NCT03274804). IL-1 inhibition has also shown encouraging effects in the clinic in patients with CRC. IL-1 inhibition has shown efficacy as a monotherapy (Table 1) as well as in combination with chemotherapy, and preclinical data suggest a myeloid-derived, IL-1-dependent tumour-promoting mechanism.^116^

Another approach targeting CRC is the use of TLR agonist and antagonist therapies. TLRs form a central regulatory unit in the defence against infectious agents and shape the behaviour or phenotype of CRC tumour cells.^117^ Two ongoing trials are currently evaluating the role of TLR agonists alone or in combination with chemotherapy. The role of vitamin D (or, more specifically, the modified vitamin D-binding protein macrophage activator EF-022) in macrophage activation is also being evaluated in clinical trials, thus potentially extending the beneficial effects of vitamin D beyond the adaptive arm of the immune system.^118^ In addition, another new avenue in the modulation of innate immune cells involves the combination therapy of atezolizumab (anti-PD-L1) with cobimetinib (MEK inhibition); it is assumed that synergistic myeloid cell modulation and parallel lymphocyte activation are induced, the precise mechanism of action in humans is not yet fully elucidated^119^ and clinical trial data has shown no effects in larger cohorts (IMblaze370 study^120^).

In contrast to these newer developments, two types of drug that have macrophage modulatory properties and a long history in medicine are bisphosphonates and trabectedin. Bisphosphonates have cytotoxic and inhibitory effects on myeloid cells, and clinical effects beyond the principal use for bone metastases have highlighted their immunomodulatory properties.^121^ Trabectedin was developed as an anti-proliferative agent but was subsequently found to induce significant monocytic cell depletion.^122^ Further research is needed to better understand the potential of these approaches in cancer therapy.

The enormous heterogeneity and plasticity of macrophages and the vast array of modulatory signals from the microenvironment together make successful immunotherapy aimed at targeting macrophages a complex and difficult approach to navigate. The omnipresence of macrophages and their power to destroy tumour cells, however, make attempts in this field of ‘myeloid-immunotherapy’ worthwhile.

### Neutrophil granulocytes

Together with MDSCs^123^ and macrophages,^124,125^ neutrophil granulocytes, which are especially enriched in CRC, form a complex network of phagocytosing and immunomodulatory immune cells.^126,127^ Similar to macrophages, difficulties in the classification and functional characterisation of these cells make directed interventions difficult; however, it is clear that multiple interventions (including GM-CSF, VEGF and chemokine inhibition) can modulate these cells and therefore alter the immunological microenvironment of the tumour. The effect of these interventions is also reflected by changes in the neutrophil-to-lymphocyte ratio, which serves as a secondary biomarker for therapy success in many clinical trials.^128^

Clinical trials (Table 1) that modulate this group of immune cells are numerous; one such example for neutrophil and MDSC targeting is the inhibition of arginase (produced by these cells), which subsequently leads to T-cell activation.^118,130–134^ Interestingly, higher densities of tumour-associated neutrophils were associated with better prognosis in CRC^134^ and, even more surprising, with a better response to fluoracil‐based chemotherapy. Nevertheless, the robust quantification and localisation of neutrophil granulocytes in tissues is still a challenge, again similar to the situation for macrophages.^135–137^

### NK cells

NK cells are a subtype of innate lymphoid cell; they are therapeutically attractive owing to their capacity to kill tumour cells without requiring further ‘education’ by other immune cells. It has become clear that there are far more regulatory (and inhibitory) mechanisms within the microenvironment of solid tumours than expected, and studies of CRC and breast cancer have identified that infiltrating NK cells can be selectively suppressed.^18,21^ Activating and inhibiting receptors, such as killer cell immunoglobulin-like receptors, together with their ligands, form an intricate network that regulates NK cells^138–141^ and consequently offer potential for translational intervention. Therefore, aside from the potential to modulate NK cell activation or inactivation in the clinic (Table 1), approaches involving cellular therapies have gained more attraction and trials are underway to evaluate the potential for NK cells in CRC. Of note, many checkpoint inhibitor therapies not only influence effector T cells but also NK cells. The pathway and magnitude of NK cell modulation (via, e.g., PD-1, 4-1BB, CD27, etc.) are poorly understood and the parameters for further combinations and selection of defined patient cohorts are therefore being evaluated.^142,143^

### Fibroblasts

Besides their structural role in tissues, fibroblasts also have a fundamental immunological role, especially with respect to modulation of the innate immune system.^144,145^ Their inflammatory potential together with their orchestrating function (e.g., via chemokines) make these cells an important immunologic interface. Current clinical trials are either aimed at the destruction (e.g., by targeting fibroblast-activating protein α) or the modulation of fibroblast function; the latter can be achieved by modulating key signalling pathways, including those involving fibroblast growth factor, platelet-derived growth factor, or stromal-derived factor 1α/CXCL12. Clinical trials in overlapping functional areas (e.g., inhibition of angiogenesis and stromal modulation) are common; afatinib provides an example of this, as it targets the stromal compartment and stroma formation. Furthermore, CXCL12 inhibition in cancer-associated fibroblasts showed effects in preclinical models,^146^ with results indicating that modulation of this axis would abrogate anti-migratory effects, leading to an influx of T cells and tumour cell attack.

## The microbiome and modulation of innate immunity in CRC

Survival of the human body depends on tight control of the microbiota, particularly in the gut, and the prevention of unwanted infections. Intestinal epithelial cells are equipped with a vast array of innate immune receptors, highlighting the intimate interplay between the gut content and the immune system.^147^ Furthermore, signalling by TLRs—among other molecules—is an important pathway in regulating innate immune activation and involves proteins such as MyD88, TNF-associated factor 6 and nuclear factor-κB.^148,149^ Dysregulation of this pathway can lead to autoimmunity (e.g., colitis or chronic IBD) or neoplastic transformation.^9,150,151^

Alterations in the composition and localisation of distinct bacterial species within the gut can disturb the equilibrium with the innate immune system. Certain bacteria (e.g., *Helicobacter hepaticus*) can promote carcinogenesis directly by producing reactive oxygen species, whereas others (e.g., *Fusobacterium nucleatum*) induce complex immunomodulation that supports the tumour.^152–154^ Furthermore, it was recently shown that the microbiome can shape the response to immunotherapy.^155–157^ The effects of the microbiome on the adaptive arm of the immune system have been described extensively, but very little is known about the bacterial species, effector molecules and molecular regulation through which the microbiome modulates the innate arm of the immune system.^158^ As described above, there has so far been limited success in immunotherapy for CRC and our understanding of the microbiome and its therapeutic potential in altering the innate immune system is still in its infancy. However, one approach includes the application of probiotics to modify the composition of the bacterial species that are present in the gut of cancer patients and thereby not only ‘correct’ the microbiome but also induce favourable clinical effects for immunotherapies or the course of the disease altogether. This attempt is extremely complex due not only to the lack of a definition of a ‘beneficial’ microbiome for an individual patient, but also due to technical issues of (prolonged) ‘implantation’ of a new microbiome into a patient.^159^ This approach has therefore only reached entry level for clinical use.^160–162^ It remains to be seen how these observations can be exploited for CRC.

## Optimisation of immunotherapy: innate and adaptive immunity together

Careful analyses of the immunological parameters of the local microenvironment have revealed the presence of multiple complex regulatory systems at the tissue level.^46,163–166^ The local microenvironment in different organ sites, particularly in metastatic disease, needs to be targeted specifically to enable immunotherapy to be successful. Furthermore, data from clinical trials and limited preclinical models underscore the interdependency between the innate immune system and the adaptive immune response. We need to ‘reprogramme’ the innate immune system, in order to allow long-lasting effector-lymphocyte tumour cell killing; to reach this stage, a greater understanding is required of the tissue-level complexities for the underlying immunological mechanisms, including migration, differentiation, plasticity, adjuvanticity and anti-tumoural functionality. These interdependent systems within the tissue require careful analysis and an improvement in our understanding of the dynamics behind the situations we observe in the clinic.

The role of interventions in the preventive setting also need to be better understood, with data from the systematic use of aspirin and other medications, suggesting a preventive role for certain medications in inhibiting tumour growth and initiation via modulation of immunological parameters.^167,168^ Yet, given the abundance of clinical evidence, the use of aspirin and its modulatory role in established CRC are not reflected in the current trial landscape, which is an obvious paradox. To escape this shortcoming, a better understanding of the complexities of the immunobiology of (metastatic) CRC with implications for therapeutic combinations and decision making is paramount. Metastatic CRC is not a disease of one system; rather, it comprises multiple diseased systems within a patient and better tools—including multiplex imaging, proteomics, computer modelling^169^ and others—are needed to fully understand the underlying networks.^170,171^ The development of parallel links between early-phase clinical trials and biopsy tissue samples is an emerging aspect; given the differences between the biology of the innate immune system in humans and in animals, analysis of human material from clinical trials will be fundamental in ensuring successful therapeutic developments.

## Conclusion

The adaptive and innate arms of the immune system are interlocking systems, tightly regulated to protect the human body and maintain integrity, and influencing all possible aspects of cellular regulation; immunological pathways are only one aspect of this regulation. In metastatic disease particularly, we observe a highly specialised network of exploitation, with selective pressure leading to this new cellular composition at the metastatic site. Far from supporting the patient, this microenvironment is optimised for survival of the tumour cells and any interventions will need to overcome the specific regulatory networks responsible. Our existing understanding of the innate arm of the immune system needs to be improved rapidly to devise synergistic and effective clinical strategies. For immunotherapy in solid (metastatic) tumours, synergies between the adaptive and innate arms of the immune system can clearly be harnessed to enhance the anti-tumoural response. In this setting, the precise regulation and timing that govern the activation of the innate immune system are still poorly understood. Data from animal models and clinical trials have indicated an obvious need to better understand the intricate networks of the innate immune system in different affected organs and at different time points during the disease (e.g., localised disease vs. progressive metastatic disease). New models might help to understand the intricacies of the different cellular phenotypes of innate immune system components; understanding the local composition of these cells is key for the application of strategies that target the innate arm as successful immunotherapies in the clinic.
